# The structural basis of nanobody unfolding reversibility and thermoresistance

**DOI:** 10.1038/s41598-018-26338-z

**Published:** 2018-05-21

**Authors:** Patrick Kunz, Katinka Zinner, Norbert Mücke, Tanja Bartoschik, Serge Muyldermans, Jörg D. Hoheisel

**Affiliations:** 1 Division of Functional Genome Analysis, German Cancer Research Center (DKFZ), Im Neuenheimer Feld 580, 69120 Heidelberg, Germany; 2 Division of Biophysics of Macromolecules, German Cancer Research Center (DKFZ), Im Neuenheimer Feld 580, 69120 Heidelberg, Germany; 3 NanoTemper Technologies GmbH, Flößergasse 4, 81369 Munich, Germany; 4 Laboratory of Cellular and Molecular Immunology, Vrije Universiteit Brussel, Pleinlaan 2, 1050 Brussels, Belgium

## Abstract

Nanobodies represent the variable binding domain of camelid heavy-chain antibodies and are employed in a rapidly growing range of applications in biotechnology and biomedicine. Their success is based on unique properties including their reported ability to reversibly refold after heat-induced denaturation. This view, however, is contrasted by studies which involve irreversibly aggregating nanobodies, asking for a quantitative analysis that clearly defines nanobody thermoresistance and reveals the determinants of unfolding reversibility and aggregation propensity. By characterizing nearly 70 nanobodies, we show that irreversible aggregation does occur upon heat denaturation for the large majority of binders, potentially affecting application-relevant parameters like stability and immunogenicity. However, by deriving aggregation propensities from apparent melting temperatures, we show that an optional disulfide bond suppresses nanobody aggregation. This effect is further enhanced by increasing the length of a complementarity determining loop which, although expected to destabilize, contributes to nanobody stability. The effect of such variations depends on environmental conditions, however. Nanobodies with two disulfide bonds, for example, are prone to lose their functionality in the cytosol. Our study suggests strategies to engineer nanobodies that exhibit optimal performance parameters and gives insights into general mechanisms which evolved to prevent protein aggregation.

## Introduction

**Figure 1:**
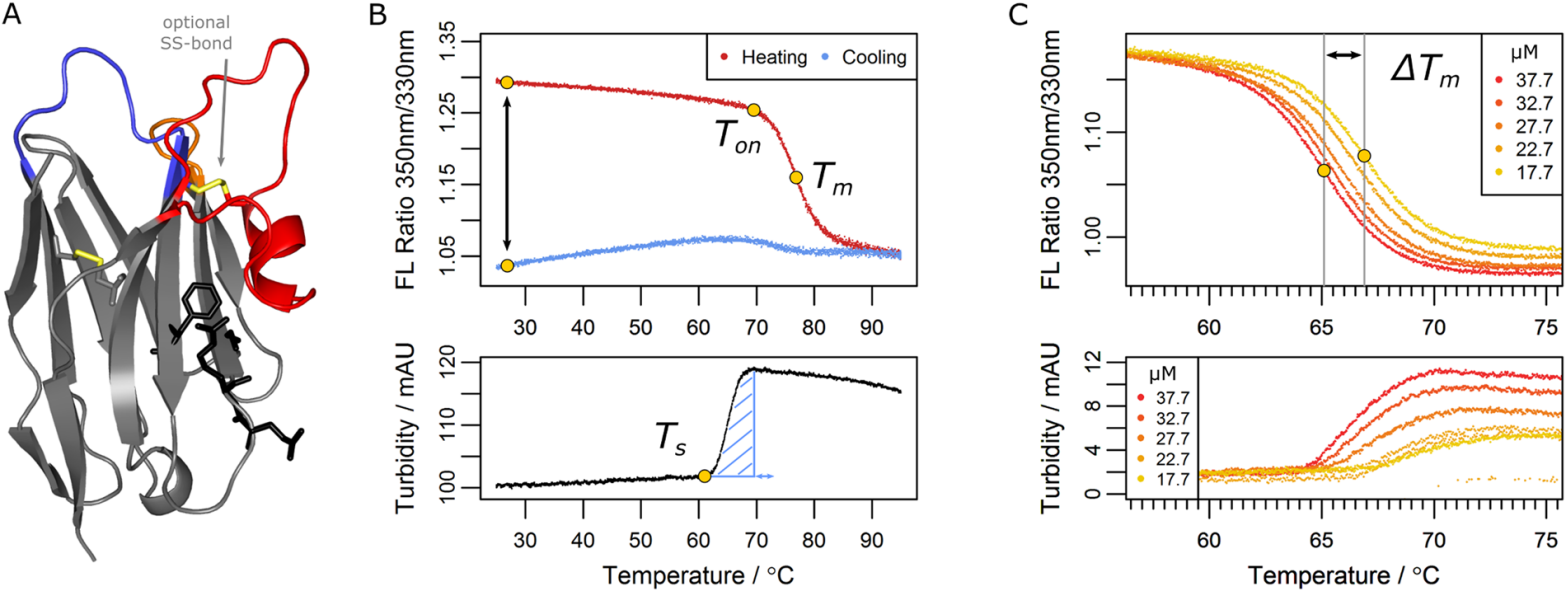
Parameters determined in the nanobody analysis. (**A**) A typical nanobody scaffold is shown (PDB ID: 1MEL). CDR loops are highlighted: CDR1, blue; CDR2, orange; CDR3, red. Hallmark positions are shown as black sticks, conserved and optional disulfide bonds as yellow sticks. (**B**,**C**) Parameters obtained from DSF and turbidity assays scanning a temperature range of 25 °C to 95 °C. (**B**) Upper panel: The ratio of intrinsic protein fluorescence emission (350 nm/330 nm) reports about the onset temperature of unfolding (T_on_) and the melting point (T_m_) during the heating phase. A difference of zero between initial and final ratio values after a complete temperature cycle (black arrow) would indicate complete reversibility. Lower panel: The turbidity trace of the heating phase yields the onset temperature of aggregation (T_s_) and the turbidity integral (blue shaded area); the latter serves as a qualitative measure of aggregation. If T_s_ occurs during the cooling phase, the turbidity integral is determined in reverse orientation. (**C**) Upper panel: Apparent melting temperature (T_m_) values yield the ΔT_m_ shift when aggregation is modulated by the nanobody concentration. The ΔT_m_ shift can serve as a measure of aggregation propensity. Lower panel: the directly related turbidity traces are shown.

The antibody repertoire of camelids contains heavy-chain antibodies (HCAbs), which represent a remarkable evolutionary exception: their structure comprises two heavy chains only, lacking the additional light chains of conventional antibodies. As a result, the derived antigen-binding domain – called nanobody or VHH (variable domain of the heavy chain of HCAbs) – is a natural single-domain antibody with several unique qualities. Technologically important is their tendency to bind structured, often cryptic epitopes that are frequently inaccessible to conventional antibodies. This is due to the nanobodies’ small size of around 15 kDa and the convex shape of the paratope architecture (Fig. 1A). In combination with a third complementarity determining region (CDR3) of unusual length, they are capable of binding specifically to enzyme active sites^1,2^ and conserved epitopes of virus particles^3^, or capture transient protein conformations^4,5^. As a small, intrinsically monomeric domain, nanobodies are known to be distinctly more soluble than conventional, antibody-derived scaffolds. *In vivo*, they share a decent conformational stability and exhibit efficient tissue penetration and relatively low immunogenicity^6,7^. Specific binders are easily selected, manipulated and produced in large amounts using standard recombinant techniques. Expectedly, a continuously growing array of applications in research, diagnostics and therapy emerged in recent years that uses nanobodies in cell biology^8^, structural biology^9^, for super-resolution microscopy^10,11^, as diagnostic agents^12^ or potent inhibitors of cancer-associated proteins^13^.

Soon after the discovery of HCAbs in 1993^14^, several studies pointed at superior biophysical properties of the nanobody binder class^15,16^. While their thermodynamic stability turned out to be comparable with conventional VH domains^17^, several reports suggested that the reversibility of nanobody denaturation represents the most remarkable difference to conventional binders^15,17,18^. It seemed to be based on a simple two-state mechanism of folding, devoid of intermediate states and reversible even upon heat-denaturation^19^. If heat-induced irreversible inactivation of nanobodies was observed, it was suggested to be due to chemical modifications of amino acids and not due to aggregation^20,21^. These superior examples of nanobody binders tend to shape the conception of nanobody thermoresistance in the literature^4,6,12^. However, they were contrasted by several reports about nanobody aggregation which is dependent on environmental conditions^16,22–24^ and inspired the development of mutational strategies to improve their thermoresistance and refolding behavior^25–27^. First, a major goal of this study is to define a quantitative model of nanobody thermoresistance that clarifies this controversy. Second, the molecular determinants responsible for both aggregation as well as reversible refolding remain poorly defined and are investigated in our work to enable their engineering.

Several mechanisms have been identified that prevent protein aggregation and contribute to reversibility of protein unfolding in general. Besides negative design elements^28^, shielding of aggregation-prone patches^29,30^, charge effects^28^ and fine-tuned structural dynamics^31,32^, kinetic barriers emerged as an important reason for reduced protein aggregation propensities^33,34^. Most strategies to avoid aggregation aim at circumventing aggregation-prone conformations^35^. Similarly, two characteristics were considered to explain the solubility of natively folded nanobodies. First, there are four hallmark positions which mediate the dimerization between VH and VL domains in conventional binders but are mutated to slightly more hydrophilic amino acids in nanobodies (Fig. 1A)^36,37^. Second, the CDR3 loop usually forms a small hydrophobic cluster below its N- and C-terminal boundaries, which was shown to contribute to nanobody stability^38^ but could also prevent nanobody dimerization by partly covering the former VH-VL interface.

Here, we present a quantitative characterization that defines nanobody thermoresistance and aggregation behavior in unprecedented detail. It allows defining unknown principles of nanobody thermoresistance and their unfolding reversibility. In addition, it offers knowledge that is important for the selection and engineering of nanobodies. It also illustrates the potential of high-throughput protein stability measurements to generate information to such ends. By quantifying the melting behavior in thermal scans for almost 70 nanobodies under various conditions, we found that irreversible aggregation plays a considerable role in heat-induced nanobody denaturation. Concentration-dependent T_m_ measurements yielded a measure of nanobody aggregation propensity. It also indicated that an additional disulfide bond is a protective factor against nanobody aggregation, fostering their reversibility. Its effect is particularly pronounced in combination with a long CDR3 loop, further suggesting that an effective shielding of the former VH-VL interface is a prerequisite for nanobody thermoresistance and folding reversibility.

## Results

### Irreversible processes are a substantial part of heat-induced nanobody denaturation

The nanobody scaffold has been reported repeatedly to reversibly refold after heat-denaturation, apparently devoid of aggregation, a view that is contrasted by several examples of aggregating nanobody binders. To properly define the thermoresistance of the nanobody fold in general and to reveal the molecular basis of both nanobody aggregation and reversible refolding, we characterized 68 affinity-matured, dromedary- and llama-derived nanobodies employing differential scanning fluorimetry (DSF) and parallel turbidity assays. Relevant parameters were obtained as illustrated in Fig. 1B. Performing measurements at nanobody concentrations of 13.1 and 32.7 µM (corresponding to around 0.5 and 0.2 mg/ml) yielded a multidimensional data set on nanobody thermoresistance for a heating rate of 0.5 °C/min.

**Figure 2:**
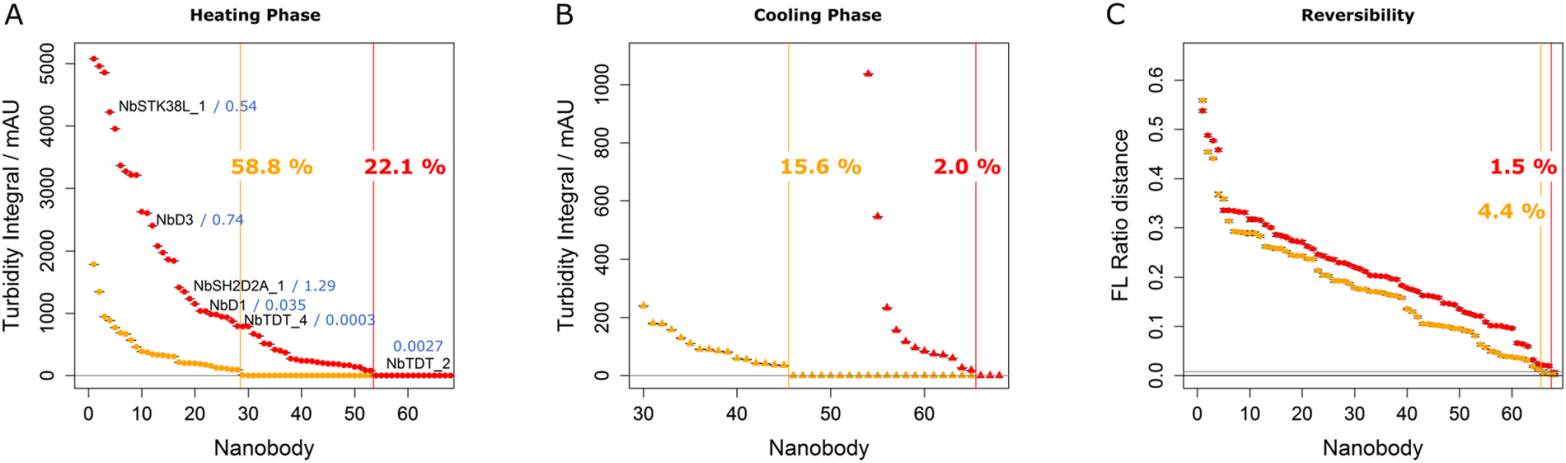
Fraction of aggregation-free nanobodies. (**A**) Size-ranked turbidity integrals obtained during the heating phase. Integrals for nanobodies devoid of a significant turbidity signal were set to zero (data points to the right of the colored, vertical lines; red: 32.7 µM, orange: 13.1 µM). Data points in a single column refer to the same nanobody. Name-labeled data points correspond to the kinetically characterized nanobodies of Fig. 3A with aggregation rate constants in blue and in s^−1^. For error calculation see Methods section; for raw traces see Supplementary Figure 1. (**B**) Turbidity integrals as in panel A but for the cooling phase. The percental fractions of aggregation-free nanobodies refer to the full set of 68 binders. For raw traces see Supplementary Figure 2. (**C**) Fraction of reversibly refolding nanobodies judged from differences between initial and final fluorescence ratios. For calculation see Methods section. Values for 32.7 (red) and 13.1 µM (orange) were separately ranked by size. Horizontal gray line: Threshold of significance (three times the average standard error observed in initial and final intervals of a 2 °C range).

First, we determined the fraction of aggregation-free nanobodies by means of turbidity assays. To ease interpretation, turbidity signals were integrated over a temperature range of 7 °C above the respective onset temperature of aggregation (T_s_) (Fig. 2A; for raw traces see Supplementary Figure 1). At 32.7 µM, only 22.1% of the investigated binders were devoid of significant aggregation during the heating phase to 95 °C. At 13.1 µM, this fraction increased to 58.8%, indicating a strong concentration dependence of turbidity signals. When including the data of the cooling-phase (Supplementary Figure 2), the fractions of zero turbidity dropped to merely 2.0% at 32.7 µM and 15.6% at 13.1 µM, suggesting the presence of at least some aggregation for a substantial percentage of nanobodies.

Second, we measured the fraction of reversibly refolding nanobodies by comparing fluorescence ratio values prior and after a complete heating and cooling cycle (Fig. 2C). After one cycle at 32.7 µM, only 1.5% of the binders fully recovered the initial fluorescence level, increasing to 4.4% at 13.1 µM. While these numbers might be biased due to unequilibrated refolding reactions for some binders^39^, they are in basic agreement with the aggregation data obtained in the turbidity assays (Fig. 2A and B). Furthermore, melting curves for two representative nanobodies were monitored using CD spectroscopy, showing that merely 1% and 43% of the folding amplitude were recovered after a full temperature cycle for nanobodies NbD3 and NbD1, respectively (see Supplementary Figure 3 and compare their turbidity integrals in Fig. 2A). These data clearly confirmed that for a large fraction of nanobodies, irreversible processes take place upon heat-induced denaturation.

### Aggregation is the major source of irreversibility of heat-induced nanobody denaturation

**Figure 3:**
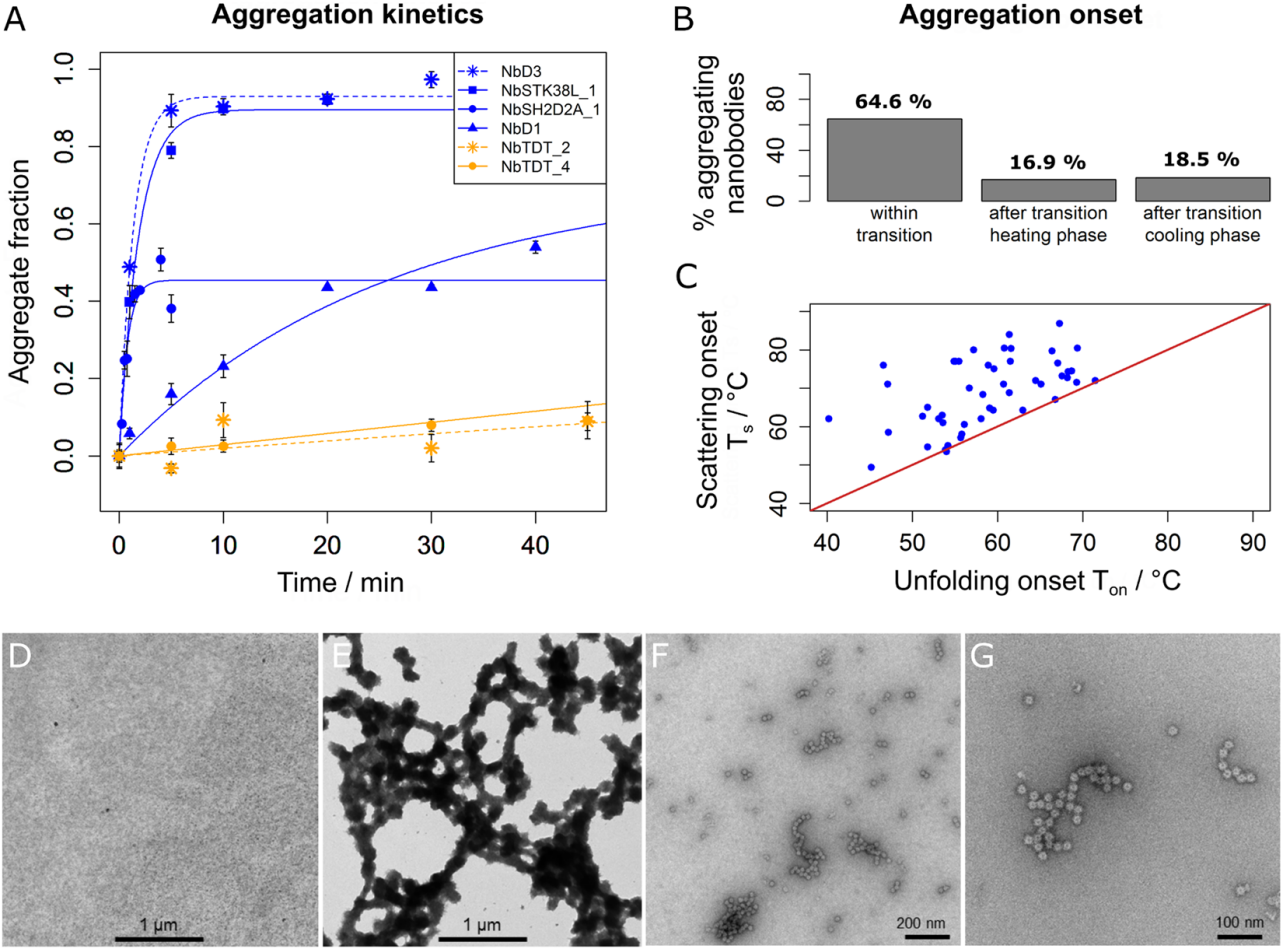
Nanobody aggregation kinetics, structure and mechanism. (**A**) Monomer loss was monitored in centrifugation assays by separating aggregates from the soluble nanobody fraction at various time points. Measurements were performed in triplicate at the T_m_ value of each nanobody at a concentration of 32.7 µM. Dashed and solid lines represent single exponential fits. Fit parameters and T_m_ values are given in Supplementary Table 1. Orange-labeled nanobodies contain two disulfide bonds, blue labeled nanobodies exhibit one disulfide bond. (**B**) Distribution of aggregation onset temperatures T_s_ over different temperature regimes. (**C**) Relation of unfolding onset temperatures T_on_ and scattering onset temperatures T_s_ for the 64.6% of nanobodies aggregating within the unfolding transition of the heating-phase. The large majority of points lies above the diagonal, indicating that aggregation requires nanobody unfolding. (**D**–**G**) Electron micrographs of nanobody NbD1 aggregation at 32.7 µM: (**D**) Native protein; (**E**) fully aggregated protein after 30 min at 90 °C; (**F**,**G**) status after 35 min at the T_m_ value of NbD1 (65.4 °C).

To attribute these observations to the occurrence of protein aggregates, the kinetics of monomer loss was measured for six nanobodies that were picked from across the entire range of turbidity integrals in Fig. 2A. They revealed a broad range of aggregation rates covering several orders of magnitude (Fig. 3A). Also, the presence of a threshold concentration was shown, which was required for aggregation in case of NbSH2D2A_1 (for first order rate constants and final amplitudes see Supplementary Table 1). Notably, the observed aggregation rates roughly reflected the corresponding turbidity integrals (see labels in Fig. 2A for comparison). A structural characterization using electron microscopy (Fig. 3D–G) showed that aggregated nanobodies form round-shaped particles with a diameter of around 20 nm which further crosslink to higher order aggregates upon prolonged heating, a process which we confirmed to be irreversible by showing that incubation at room temperature for 24 h did not reverse aggregation (Supplementary Figure 4).

For each nanobody, we determined the temperature regime of the respective aggregation onset, revealing that the majority of binders aggregated within the unfolding transition or at higher temperatures (Fig. 3B; see Supplementary Figure 5 for an illustration of aggregation regimes). The fact that aggregation was not detected below the onset temperature of unfolding (T_on_) indicated that aggregation requires nanobody unfolding. This is documented by (i) the comparison of T_s_ and T_on_ values (Fig. 3C), (ii) the detection of highly homogeneous and monomeric nanobodies using analytical ultracentrifugation (Supplementary Figure 6), and (iii) the absence of aggregates prior to heating (Fig. 3D). The last was confirmed by the exclusive detection of monodisperse protein peaks in size exclusion chromatography under such conditions (data not shown). Furthermore, apparent 1^st^ order aggregation kinetics was obtained (Fig. 3A), a phenomenon commonly observed if protein unfolding represents the rate-limiting step in the aggregation reaction^40^. Small nanobody fractions aggregated at temperatures above the completed unfolding transition or during the cooling phase.

In summary, these results underlined the remarkable solubility of native nanobodies but confirmed that irreversible aggregation is a serious phenomenon in heat-induced nanobody denaturation for the majority of binders.

### The ΔTm shift as a numeric description of nanobody aggregation propensity

Particularly in a therapeutic context, non-native protein aggregation poses a serious risk, e.g. triggering immune responses in patients^41,42^. Therefore, it is of considerable interest to study its determinants for avoiding risk factors or potentially restoring nanobody folding reversibility through protein engineering. However, a comparison of aggregation propensities among a set of binders as diverse as ours remains challenging^43,44^. Turbidity signals are qualitative due to their dependence on aggregate size and shape^44^. Therefore, we approached a quantification of nanobody aggregation propensities by means of apparent T_m_ values, which are solely a function of kinetic parameters. Following Le Chatelier’s principle^45^, aggregation of unfolded nanobodies will cause the folding equilibrium to shift towards the unfolded state, reflected in apparently decreased T_m_ values in thermal scans^46^ as demonstrated (Fig. 1C). Aggregation rates are strongly concentration-dependent, allowing a modulation of this reaction and thus an investigation of its effect on T_m_. Two factors influence the shift of apparent T_m_ values: the intrinsic aggregation rate and the kinetic stability of a nanobody^33,34,47^. The higher the latter the more inert is the folding equilibrium towards aggregation, resulting in smaller shifts.

**Figure 4:**
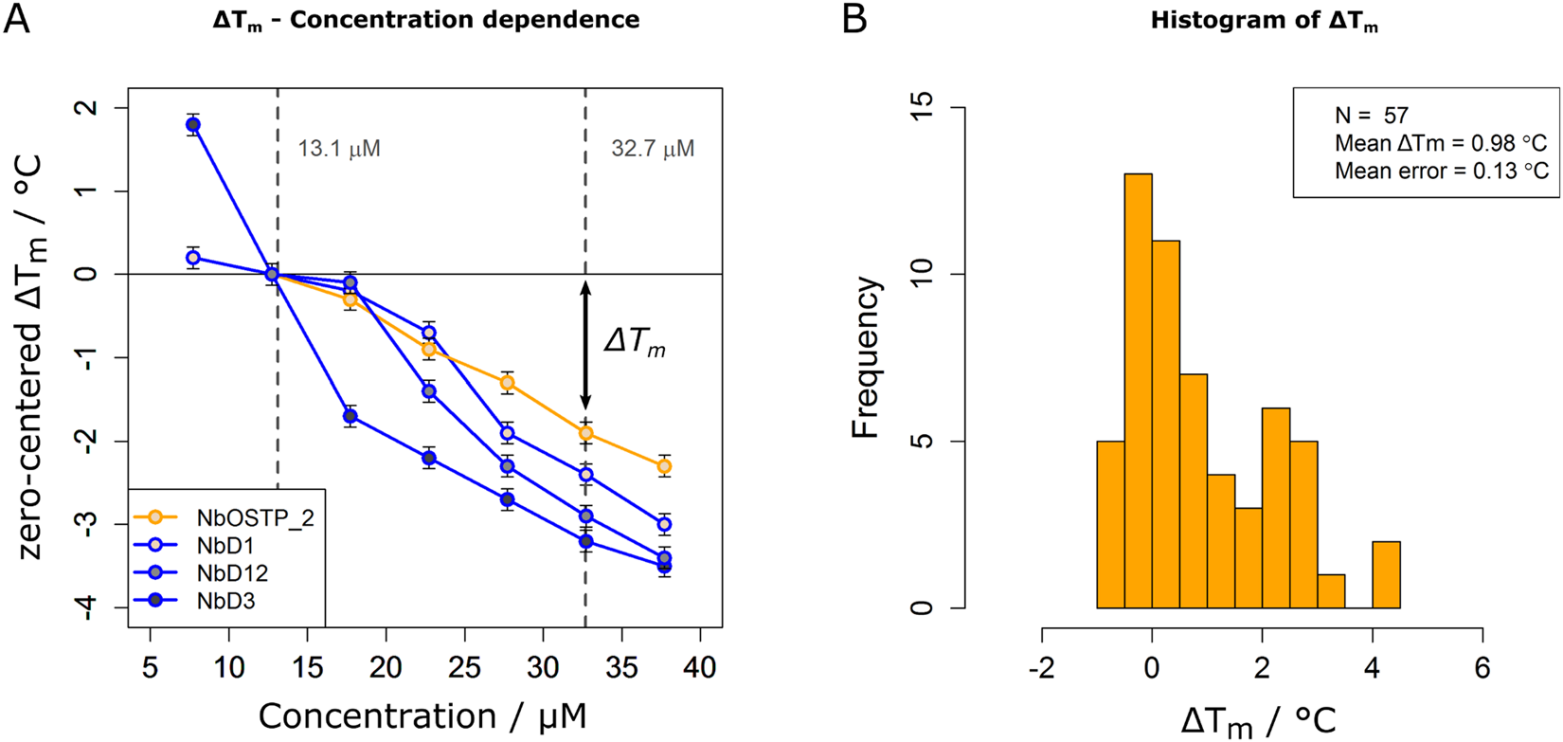
Characterization of the ΔT_m_ shift. (**A**) Concentration dependence of apparent T_m_ values measured for four nanobodies and plotted in a zero-centered fashion around the T_m_ values at 12.7 µM; orange curve: nanobody with a second disulfide bond; blue curves: nanobodies devoid of a second disulfide bond. Vertical dashed lines indicate the concentrations used in the experiments described before. Error bars represent the average standard error of 0.13 °C for a T_m_ measurement. (**B**) Histrogram of ΔT_m_ values calculated for 57 nanobodies using the equation ΔT_m_ = T_m_(13.1 µM) − T_m_(32.7 µM). 11 nanobodies were excluded since at one of the two concentrations a T_m_ value could not be unambiguously assigned.

By relating apparent T_m_ values at two concentrations, a simple measure of this effect is obtained, which we called ΔT_m_ shift. To characterize it, apparent T_m_ values of four nanobodies were determined as a function of concentration (Fig. 4A). The observed curve shapes ranged from hyperbolic to nearly linear for NbD3 and NbOSTP_2, respectively, indicating various susceptibilities of the folding equilibrium or different aggregation rates. The concentrations chosen in the data set appeared to be ideal to cover different shapes, which are reflected in the resulting ΔT_m_ values. Furthermore, the results indicated that the concentration range chosen to calculate the ΔT_m_ shift has an impact on its amplitude: strongly aggregating nanobodies, showing a hyperbolic concentration-dependence of apparent T_m_ values, like NbD3 in Fig. 4A, tend to be underestimated. Nevertheless, as a simple numeric parameter, ΔT_m_ allows to statistically compare aggregation propensities within large data sets. With an average ΔT_m_ shift of 0.98 °C in our set of nanobodies (Fig. 4B) and an average standard error of 0.13 °C for individual T_m_ values, DSF measurements were sensitive enough to reliably determine the ΔT_m_ shift.

### The second disulfide bond and its role in nanobody aggregation

Using the ΔT_m_ shift, we investigated the role of an optional second disulfide bond, a characteristic structural feature of some nanobodies, and its effect on aggregation. For this, the analysis was limited to dromedary-derived nanobodies (n = 50), as a second disulfide bond was not observed in any of the llama-derived binders of our set. Second, a quantification of ΔT_m_ is meaningful only for those nanobodies, which aggregate within the temperature range of their unfolding transition. Only in this case, aggregation can affect the folding equilibrium. Nanobodies that aggregated outside of this temperature range were excluded.

**Figure 5:**
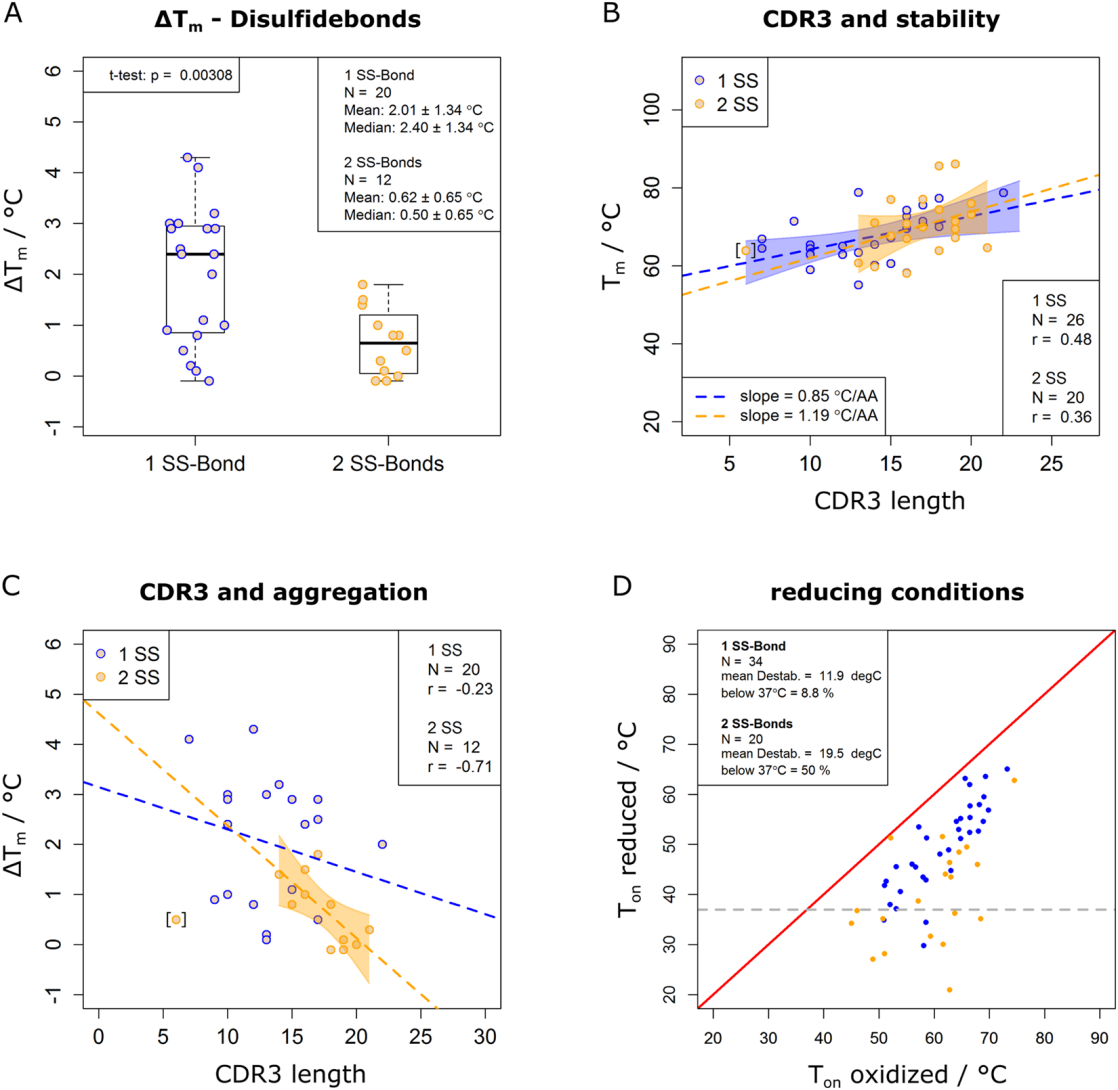
Structural determinants of nanobody thermoresistance. (**A**) A second disulfide bond (SS) suppresses aggregation. Comparison of ΔT_m_ shifts between dromedary-derived nanobodies with one and two disulfide bonds. ΔT_m_ shifts were determined at 13.1 and 32.7 µM. The p-value refers to an unpaired t-test. (**B**) The CDR3 loop length contributes to nanobody thermostability. Relation of CDR3 loop lengths and T_m_ values measured at 13.1 µM. Average CDR3 lengths are 14 and 17 amino acids for nanobodies with one and two disulfide bonds, respectively. Dashed lines represent the linear regressions in the respective group-specific color; the 95% confidence interval is indicated by a colored band. Pearson correlation coefficients (r) are shown. One nanobody with two disulfide bonds was excluded from analysis (data point in brackets) because of its unusually short CDR3 length of 6 amino acids. (**C**) A long CDR3 loop suppresses aggregation in presence of a second disulfide bond. The correlation of CDR3 loop length and aggregation propensity was measured by ΔT_m_ at 13.1 and 32.7 µM. Color codes and statistical parameters are as in panel A. (**D**) Nanobodies with a second disulfide bond are prone to lose their structure under reducing conditions. Relation of T_on_ values of nanobodies with one (blue) and two (orange) disulfide bonds in presence and absence of 25 mM TCEP, measured at 13.1 µM and a heating rate of 0.5 °C/min. A gray dashed line indicates a temperature of 37 °C.

Interestingly, a comparison of ΔT_m_ values indicated a significantly reduced aggregation propensity in presence of a second disulfide bond in dromedary-derived nanobodies (Fig. 5A, p = 0.0031). The binders chosen for measuring the kinetics of aggregation and the concentration dependence of ΔT_m_ clearly supported this effect: nanobodies with two disulfide bonds had the slowest aggregation rates (orange traces in Fig. 3A) and the least concentration dependence of ΔT_m_ (orange trace in Fig. 4A). Although a quantitative treatment of turbidity integrals needs to be handled with care, they were compared between both nanobody groups (including all dromedary nanobodies with a significant scattering onset T_s_). While not significant at 13.1 µM, turbidity integrals were clearly lower for nanobodies with two disulfide bonds at 32.7 µM (p = 0.006, Supplementary Figure 7), supporting the significance of the above finding based on ΔT_m_ analysis. We concluded that besides its well established effect on conformational stability of nanobodies (Supplementary Figure 8) and its contribution to binding affinity^48^, a third function of the second disulfide bond is to reduce nanobody aggregation, which could be due to an increase in kinetic stability, a reduction of the intrinsic aggregation rate, or due to a mixture of both effects.

### Nanobody thermoresistance is a function of CDR3 length

Notably, the second disulfide bond in nanobodies is commonly believed to rigidify and stabilize CDR3 loops which are particularly long^48,49^. If conformational stability is considered, long flexible loops are expected to be destabilizing^48,50–52^. However, their role in protein aggregation is more diverse: dynamic regions were shown to foster both aggregation^29,31^ and protein solubilization^32^. Therefore, it was interesting to ask if CDR3 loop length was somehow correlated with nanobody thermostability and aggregation behavior in presence and absence of a second disulfide bond. Surprisingly, a moderately positive correlation rather than the expected negative correlation of CDR3 length and nanobody thermostability was found both for nanobodies with one and two disulfide bonds (Fig. 5B). Interestingly, the slight trend to higher stability with increasing CDR3 length was more significant in nanobodies with only one rather than two disulfide bonds. Considering the numerous factors which govern protein stability^53,54^, it is not surprising that such a positive trend remains moderate. The result strongly contrasted the expected destabilization caused by a long and flexible CDR3-loop, if it is not stabilized by an additional disulfide bond^48,50–52^.

Similarly, the relationship of CDR3 loop-length and nanobody aggregation propensity was investigated using ΔT_m_ shifts. Strikingly, while no correlation in nanobodies devoid of a second disulfide bond was detectable (r = −0.23), CDR3 length and ΔT_m_ shifts are negatively correlated for nanobodies with two disulfide bonds (r = 0.71, Fig. 5C), suggesting that nanobody aggregation can be effectively reduced by a particularly long CDR3 loop, which is additionally stabilized by a second disulfide bond.

### Reducing conditions challenge nanobody thermostability and their application as intrabodies

The multifunctional role of the second disulfide bond – fostering conformational stability of nanobodies and contributing to binding affinity^48^ as well as reducing aggregation – might prove problematic for applications under reducing conditions, such as expressing nanobodies in the cellular cytoplasm for microscopy or functional studies^12^. To address this, nanobody stability was investigated by DSF measurements in presence and absence of 25 mM of the mild reducing agent tris(2-carboxyethyl)phosphine (TCEP). While the expected destabilization did not lower the T_m_ below a critical temperature of 37 °C in most cases (Supplementary Figure 9), Fig. 5D indicates a 50% chance that the folding equilibrium of a nanobody with two disulfide bonds is significantly shifted towards the unfolded state at physiological conditions. This result can serve as a guideline for nanobody selections and could explain observations of activity loss for a significant number of nanobodies upon cytoplasmic expression^55^.

## Discussion

Nanobodies represent an antibody-derived binder class with extraordinary potential and unique biophysical properties. Several examples of reversibly refolding nanobodies shaped this view, while nanobody aggregation and chemical modifications upon heat-denaturation were observed as well. To clarify the significance of nanobody aggregation besides reversible refolding upon heat-denaturation, we comprehensively characterized around 70 nanobodies in DSF and turbidity measurements. Our analysis defines a quantitative model of nanobody thermoresistance in which irreversible denaturation occurs to some degree for the majority of binders and is mainly caused by aggregation. Reversibly refolding nanobodies appear to represent the exception and not the rule. Chemical modifications were suggested as the dominant cause of nanobody inactivation, but are likely to play a minor or no role in our study, as they require prolonged incubation times at high temperature^20^. Despite our results, it needs to be stated clearly that, compared to conventional antibodies^56^, the general thermoresistance of nanobodies remains to be exceptional. Although using harsh conditions, a slow heating rate and high protein concentrations, a remarkable 60% of turbidity-free binders were observed at 95 °C and 13.1 µM. Furthermore, nanobody aggregation required protein unfolding, underlining the high solubility of native nanobodies. Finally, the small percentage of nanobodies that exhibited no aggregation at all and were fully reversible (Fig. 2) might be a too pessimistic result, as it was possibly influenced also by a slow refolding kinetics^39^. Nevertheless, using turbidity and centrifugation assays as well as electron microscopy, it was clearly demonstrated that aggregation plays a significant role in heat-induced nanobody denaturation, which should be considered for future applications, in particular toward therapeutic ones.

The knowledge about the extent of heat-induced aggregation further provides an opportunity of engineering nanobody stability^23^ and raises fundamental questions about its determinants. We employed the ΔT_m_ shift to identify structural features that foster nanobody reversibility. The ΔT_m_ shift is a parameter that is particularly powerful for high-throughput stability measurements: it reflects two fundamental aspects of protein aggregation (kinetic stability and intrinsic aggregation rate), is independent of aggregate size or shape, and integrates the aggregation behavior of a protein over a large concentration range. Its application is limited to proteins, however, which aggregate within the unfolding transition. Furthermore, the concentration range chosen to calculate the ΔT_m_ shift affects its amplitude differently, underestimating strongly aggregating nanobodies (e. g., NbD3 in Fig. 4A). However, the effect of stabilizing features, such as disulfide bonds or CDR3 length, are expected to be even more pronounced when expanding the concentration range towards lower concentrations, illustrating the potential of a comparison of ΔT_m_ shifts in high-throughput stability measurements. Finally, the fluorescence ratio approach used for the determination of protein melting temperatures in our study (350 nm/330 nm) was recently shown to lead to artefacts in special cases^57,58^. This usually small but possible bias is dependent on the particular shape of fluorescence spectra prior and after protein unfolding and can shift the apparently measured T_m_ value of a protein. However, the same fluorescence spectra measured at two concentrations should differ merely in amplitude but not in shape. Therefore, the ΔT_m_ shift is expected to be entirely independent of these effects, further illustrating its robustness. In contrast, the general T_m_ measurements in our study can in principle contain this bias. However, due to the large number of involved binders the conclusions obtained from our statistical analyses are not expected to be substantially affected.

Several principles of nanobody thermoresistance were revealed by this analysis. An additional disulfide bond in dromedary-derived nanobodies reduced nanobody aggregation in thermal scans. Two mechanisms can account for this phenomenon: first, the second disulfide bond could increase the kinetic stability, an ability attributed to disulfide bonds in previous studies^59–62^, resulting in a folding equilibrium with reduced susceptibility to aggregation; second, disulfide bonds were proposed to protect native proteins from dysfunctional association^63,64^ suggesting a more direct interference of the additional bond with the aggregation reaction. In both mechanistic cases, it seems highly plausible that the former dimerization interface of nanobodies plays a central role for reversibility. Protein interfaces were proposed to have an increased aggregation propensity^63^. Accordingly, a long CDR3 loop, which is additionally stabilized by an extra disulfide bond, should effectively shield the aggregation-prone interface, as observed experimentally (Fig. 5C). Furthermore, our results show (Fig. 5B) that the expected destabilizing effect of a long loop^48,50–52^ is more than compensated in nanobodies, most probably by shielding the dimerization site, thereby contributing to conformational stability. Considering these observations, it is tempting to speculate that besides an increase in sequence variability and an enlarged surface necessary for antigen binding^49^, stability and solubility were an evolutionary driving force for the development of long CDR3 loops in nanobodies.

The foregoing conclusions are based on nanobodies, which aggregated within the unfolding transition, that is about two thirds (64.6%) of all binders. Our analyses could not answer the question, if partly folded intermediates are involved within this temperature regime. Nevertheless, our observations challenge the common view of nanobodies as two-state folders^19^. In contrast, an aggregation reaction, which is independent of intermediates, seems plausible for the residual one-third fraction of nanobodies, aggregating within higher temperature regimes that are dominated by the unfolded state. Therefore, three common strategies to obtain fully reversible nanobodies are suggested: (i) favoring long CDR3 loops which are stabilized by a disulfide bond; (ii) stabilizing long CDR3 loops by other, non-covalent interactions; and (iii) solubilizing the unfolded state, for example by the introduction of repulsive charges^23,25^. Importantly, the situation changes for two scenarios: First, in applications that could involve long incubation times at very high temperatures, disulfide bonds were shown to compromise nanobody refolding ability due to heat-induced disulfide shuffling and modifications of cysteine residues^21,65^. This phenomenon can compromise the effect of disulfide bonds on thermodynamic stability and aggregation behavior. Second, applications that involve nanobody expression in the reducing environment of the cytosol strongly challenge nanobody stability, if a second disulfide bond is required. *In vivo*, nanobody folding proceeds from the reduced unfolded state. Nanobodies with two disulfide bonds should therefore be avoided in applications that include cytosolic expression or should be improved by other strategies, such as adding solubilizing tags^23,25^.

## Methods

### Expression and purification of nanobody binders

Dromedary-derived nanobody binders (n = 50) originated from different phage display screenings against various protein targets and were present in the pMECS vector coding for a C-terminal HA- and His6-tag. Llama-derived nanobodies (n = 18) were selected from a subtractive phage-display library against tissue lysates^66^ and were present in the pHEN2 plasmid coding for a C-terminal Myc- and His6-tag. Nanobodies were expressed and purified as previously described in detail^23^.

### Protein quantification

Nanobody concentration was determined spectrophotometrically at 280 nm in at least quadruplicate measurements for the initial data set shown in Figures 2 and 5, otherwise in triplicates, using sequence-based extinction coefficients^67^ and a Nanodrop ND-1000 instrument (Peqlab Biotechnologie, Erlangen, Germany).

### Differential scanning fluorimetry and turbidity assay

Differential scanning fluorimetry (DSF) measurements were performed on a Prometheus NT.48 instrument (NanoTemper Technologies, Munich, Germany) with additional back-reflection optics for determining turbidity according to the manufacturer’s instructions. To ensure equal buffer conditions, a buffer exchange was performed with all binders against a single batch of PBS, pH 7.4 using Zeba Spin desalting columns (Thermo Fischer, Waltham, USA) with a 7 kDa cut-off. Samples were centrifuged at 20,000 g for 10 mins prior their measurement and a heating rate of 0.5 °C/min was employed. Fluorescence was monitored at wavelengths of 330 nm and 350 nm using an excitation wavelength of 280 nm. Nanobody performance in a reducing environment was tested at a protein concentration of 13.1 µM by adding TCEP to a final concentration of 25 mM immediately prior to the measurement using a stock solution of 250 mM TCEP, 100 mM Tris/NaOH, pH 7.5.

### Data analysis from DSF measurements

The parameters T_m_, T_on_ and T_s_ were obtained from the Prometheus NT.48 instrument software PR.ThermControl. All fitted values were visually checked in individual fluorescence and turbidity traces to remove possible artifacts. Statistical analysis including unpaired t-tests, Pearson correlation coefficients and linear regression models were calculated using standard functions in R.

To qualitatively characterize nanobody aggregation, turbidity signals were integrated over a range of 7 °C, starting from the aggregation onset temperature T_s_. In a customized R script, the mean turbidity signal of a 2 °C range below the aggregation onset temperature T_s_ was used for baseline correction. If T_s_ occurred during the cooling phase, the turbidity integral was determined in reverse orientation, using the mean turbidity signal of a 2 °C range above T_s_ for baseline correction. The standard error $S{D}^{Int}$ of an integral was calculated by $S{D}^{Int}=\sqrt{S{D}^{2}\cdot N}$ with $SD$ being the standard error of the 2 °C range and $N$ the number of integrated data points. This procedure was performed if a scattering onset temperature T_s_ was detectable; otherwise integrals were set to zero.

To judge nanobody reversibility, fluorescence ratio differences were calculated using a customized R script. It determined the mean fluorescence ratio for the initial and final 2 °C of a temperature cycle and calculated the absolute value of their difference together with the standard error: $S{D}^{Diff}=\sqrt{S{D}_{heat}^{2}+S{D}_{cool}^{2}}$ with $S{D}_{heat}$ and $S{D}_{cool}$ as the standard errors of the 2 °C ranges of the heating and the cooling phase, respectively. The threshold of significance, which indicated non-reversibility of the folding reaction, was chosen to be three times the mean value of all observed standard errors $S{D}^{Diff}$.

### Circular dichroism measurements

Nanobody melting curves were measured between 37 °C to 95 °C at a wavelength of 203 nm in a Jasco J715 CD spectrometer equipped with a Peltier temperature control unit using a heating rate of 0.5 °C/min and a protein concentration of 15 µM in PBS, pH 7.4. Curves were fitted according to Santoro and Bolen^68^, using the values at 37 °C and 95 °C of the fits to determine respective amplitudes and calculate the signal recovery after a full temperature cycle.

### Aggregation kinetics

Nanobody monomer loss was measured in centrifugation assays at a protein concentration of 32.7 µM in PBS, pH 7.4. Nanobody aliquots were incubated at their respective T_m_ value in a PCR cycler with a heated lid. At various time points, a single aliquot was centrifuged at 22,000 g for one minute at 4 °C and the protein concentration of the soluble fraction was measured spectrophotometrically in triplicates as described above using a Nanodrop ND-1000 instrument. Assuming the loss of soluble protein to be the aggregate fraction $FA$, the data was fitted to a single exponential function: $FA(t)=A\cdot (1-\exp (-{k}_{app}\cdot t))$ where $A$ represents the final amplitude, ${k}_{app}$ the apparent aggregation rate constant and $t$ the time. For testing the reversibility of aggregation, each aliquot was split in two aliquots after heat treatment. One was immediately assayed, the second after 24 h at room temperature.

### Transmission electron microscopy

To discriminate nanobody aggregation states, protein samples were incubated for different time intervals at room temperature, T_m_, or 90 °C at a concentration of 32.7 µM in PBS, pH 7.4 and subsequently stored on ice. After loading the samples on a 300-mesh, carbon-coated grid, they were washed with PBS buffer, stained with 2% (w/v) uranyl acetate and imaged using a ZEISS EM 912 microscope with a Proscan CCD camera.

### Analytical Ultracentrifugation

Sedimentation velocity experiments were performed in a Beckman analytical ultracentrifuge (Optima XLA) in double sector aluminium centerpieces at 50,000 rpm and 20 °C. Data were collected at a wavelength of 280 nm in the continuous scan mode using a spacing of 0.003 cm. Sedimentation velocity profiles were analyzed with the software DCDT+^69^ and obtained sedimentation coefficients were corrected to standard conditions (20 °C, in water). The protein partial specific volumes were calculated from the amino acid composition to 0.714 ml/g, solvent density and viscosity was calculated through summation of the contribution of buffer components to 1.005 g/cm^3^ and 1.017 mPa*s at 20 °C using the program SEDNTERP.

### Data Availability

The datasets generated during and/or analysed during the current study are available from the corresponding author on reasonable request.

## Electronic supplementary material

Supplementary Information
