# Supplementary material for: The structural basis of nanobody unfolding reversibility and thermoresistance
Source: Sci Rep. 2018 May 21;8:7934. doi: 10.1038/s41598-018-26338-z (PMC5962586; doi:10.1038/s41598-018-26338-z)
Supplement: Supplementary file 1 — Supplementary Information [file 41598_2018_26338_MOESM1_ESM.pdf]

SUPPORTING INFORMATION

The structural basis of nanobody unfolding reversibility and thermoresistance

Patrick Kunz, Katinka Zinner, Norbert Mücke, Tanja Bartoschik, Serge Muyldermans, and Jörg D. Hoheisel

Supplementary Figures

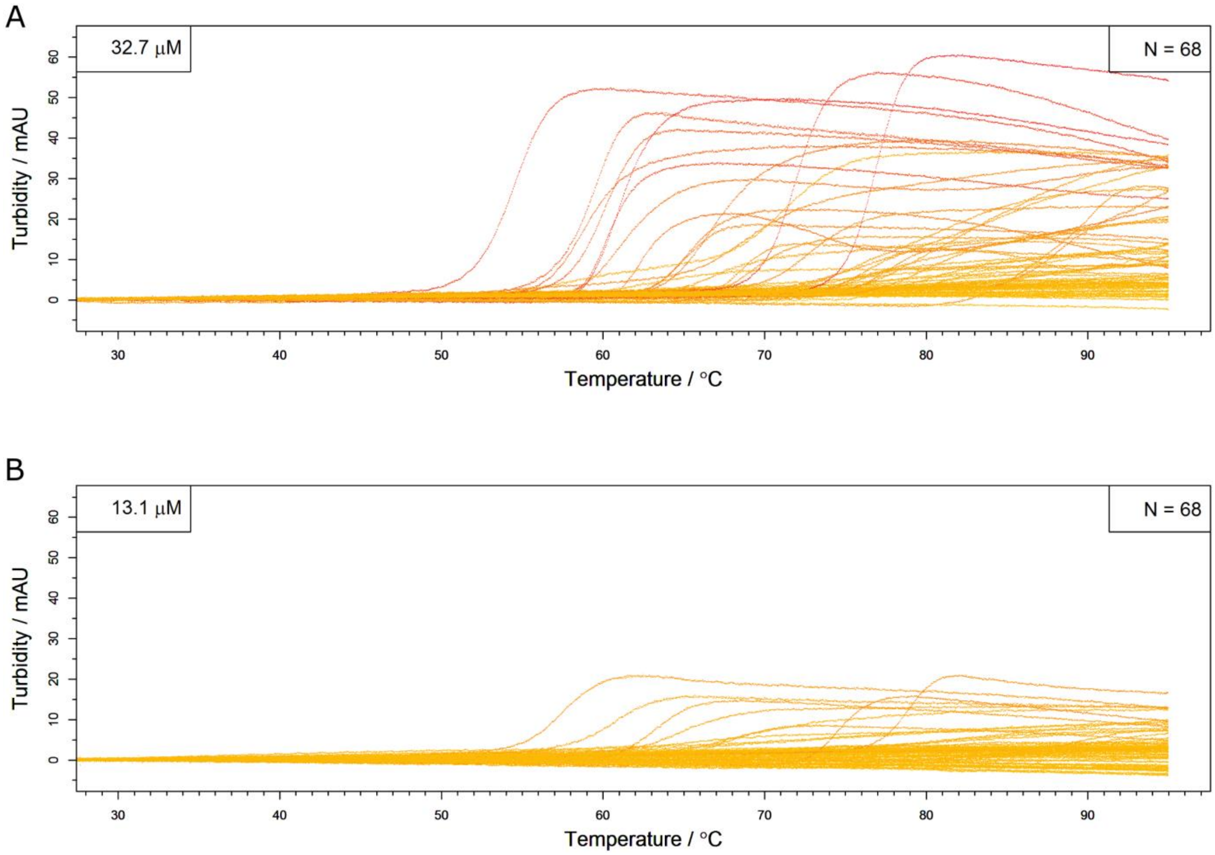

Supplementary Figure 1: Nanobody turbidity traces of the heating phase. Turbidity was measured in parallel to fluorescence measurements at 32.7 µM (**A**) and 13.1 µM (**B**) with a heating rate of 0.5 °C/min in PBS, pH 7.4. The color gradient was assigned based on turbidity integrals determined in Figure 2A of the main text with orange corresponding to lowest, red to highest turbidity.

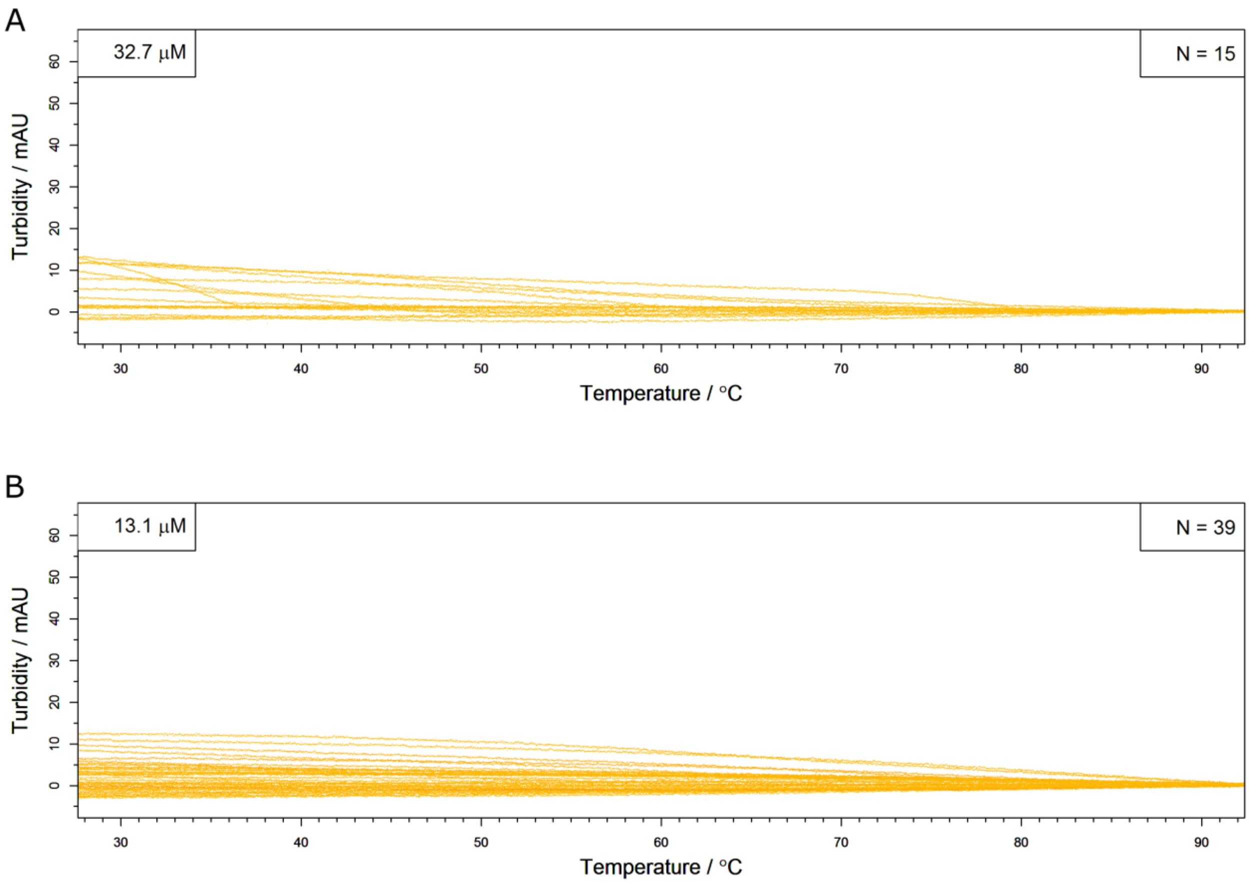

Supplementary Figure 2: Nanobody turbidity traces of the cooling phase. Turbidity was measured in parallel to fluorescence measurements at 32.7 µM (A) and 13.1 µM (B) with a heating rate of 0.5 °C/min in PBS, pH 7.4. The color gradient was assigned as in Supplementary Figure 1.

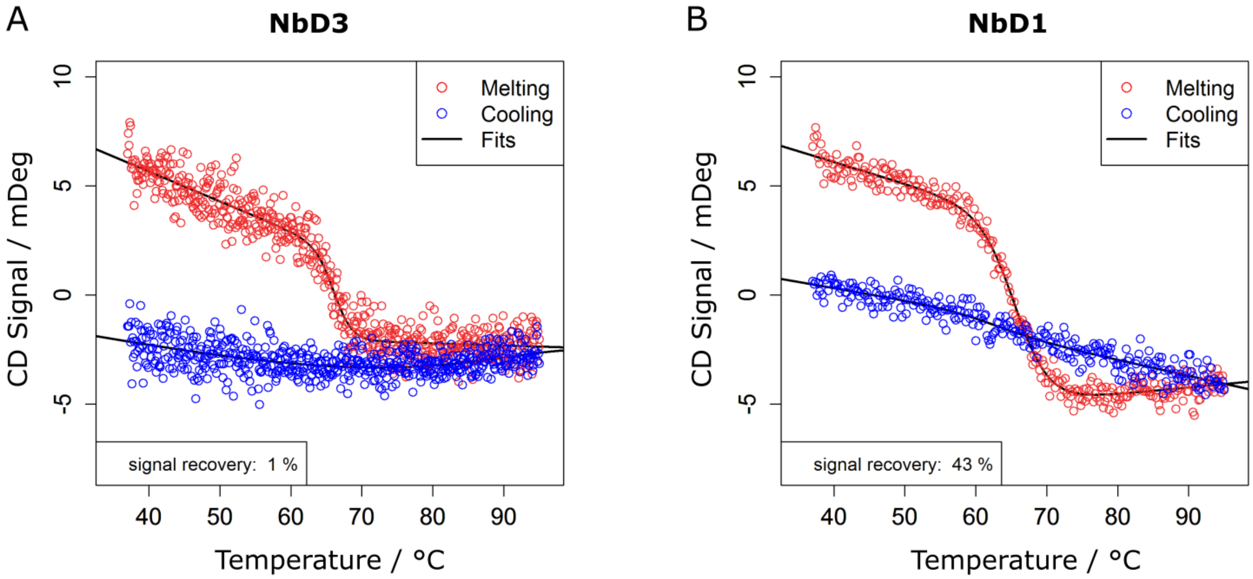

Supplementary Figure 3: Percentage of refolding after heat denaturation estimated by CD spectroscopy for representative nanobodies NbD3 (**A**) and NbD1 (**B**). Traces were recorded at a wavelength of 223 nm, using a protein concentration of 15 µM and a heating/cooling rate of 0.5 °C/min. The data pitch was 0.1°C in (**A**) and 0.2°C in (**B**). Traces were fitted according to Santoro and Bolen[61] using the values at 37°C and 95°C of the fits to determine respective amplitudes and calculate the signal recovery after a full temperature cycle.

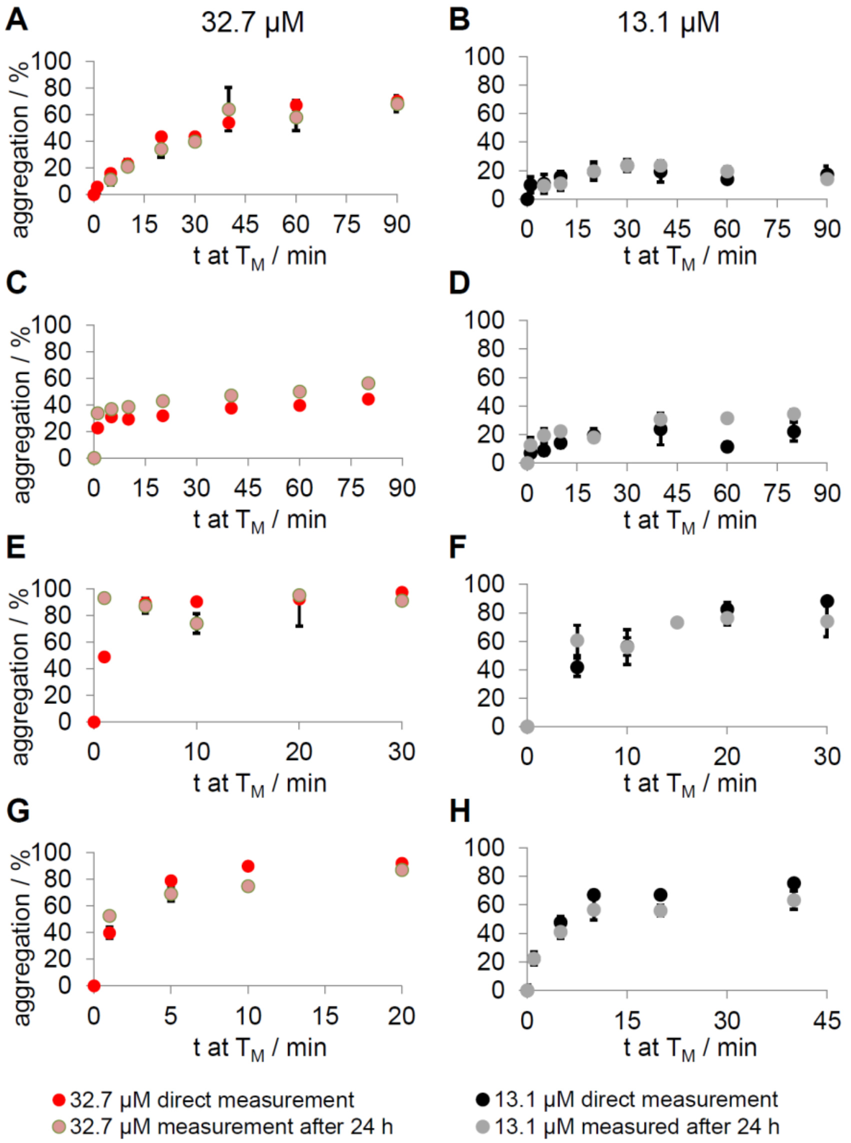

Supplementary Figure 4: Nanobody aggregation is irreversible in centrifugation assays measuring kinetics of monomer loss. Four nanobodies were heated to their respective T_m for various times at 32.7 µM and 13.1 µM, respectively. Samples were split into two aliquots: One was immediately assayed, the second after 24 hours at room temperature. Superimposing curves indicate irreversible nanobody aggregation. (**A-B**) NbD1, T_m = 65.4 µM; (**C-D**) NbD2, T_m = 62.9 µM; (**E-F**) NbD3, T_m = 67.2 µM; (**G-H**) NbSTK38L_1, T_m = 74.3 µM.

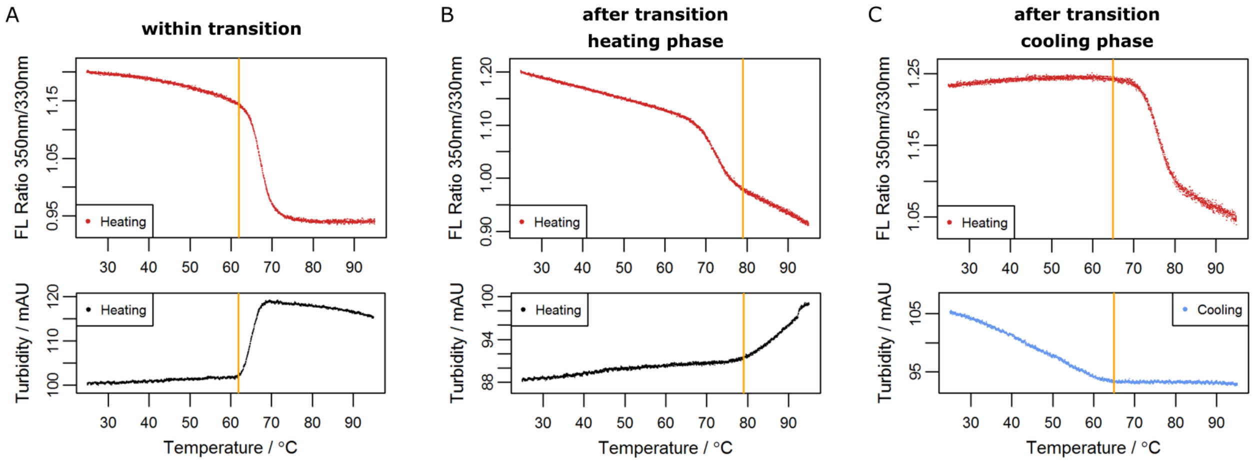

Supplementary Figure 5: Example data for the illustration of aggregation regimes. (**A**) Example traces for a nanobody aggregating within the unfolding transition. Upper panel: The ratio of intrinsic protein fluorescence emission (350 nm/330 nm) indicates the unfolding transition. Lower Panel: Corresponding turbidity trace. Orange lines indicate the scattering onset temperature T_s in both panels. (**B**) Example traces for a nanobody aggregating after the unfolding transition during the heating phase. Panels as in A. (**C**) Example traces for a nanobody aggregating after the unfolding transition but during the cooling phase.

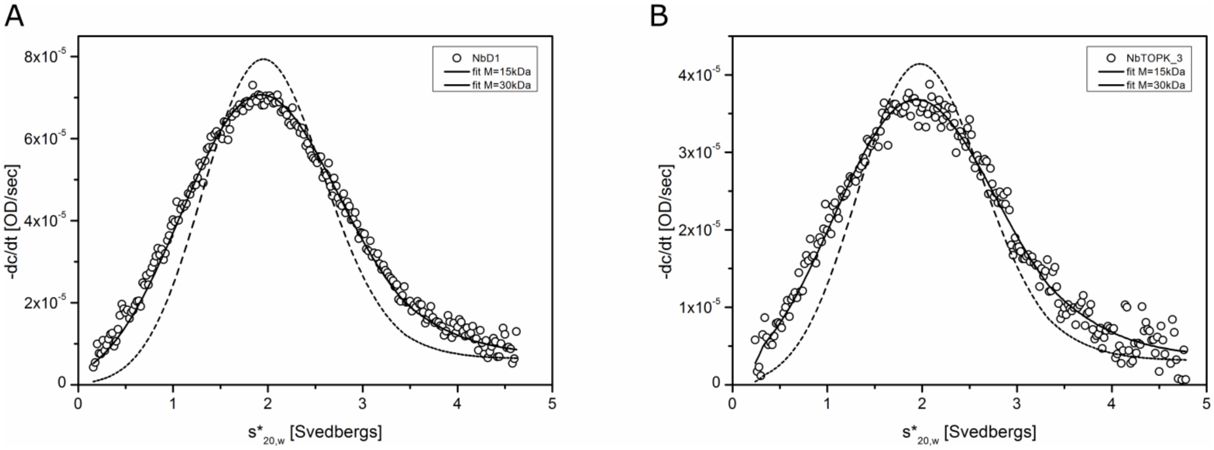

Supplementary Figure 6: Analytical ultracentrifugation of two nanobodies prior heat treatment: Sedimentation velocity profiles of NbD1 (**A**) and NbTOPK_3 (**B**). Runs were performed at a protein concentration of 0.31 mg/ml for NbD1 and 0.37 mg/ml for NbTOPK_3. The profiles were fitted to a model assuming monomers of 15 kDa (continuous lines) or dimers of 30 kDa (dashed lines), respectively, indicating highly homogenous, monomeric samples. The calculated sedimentation coefficients were 1.72 S for both proteins.

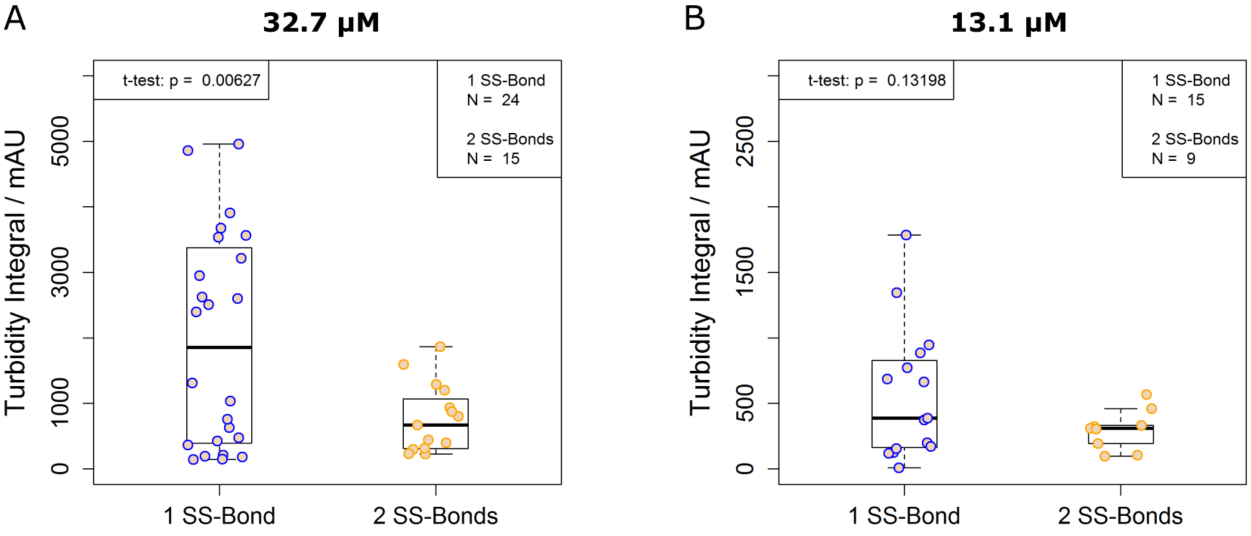

Supplementary Figure 7: Turbidity integrals of a 7 °C range for dromedary-derived nanobodies with a significant scattering onset T_S. p-values refer to an unpaired t-test. (**A**) Measurements at 32.7 µM indicate reduced nanobody aggregation in presence of a second disulfide bond. (**B**) Same measurement at 13.1 µM.

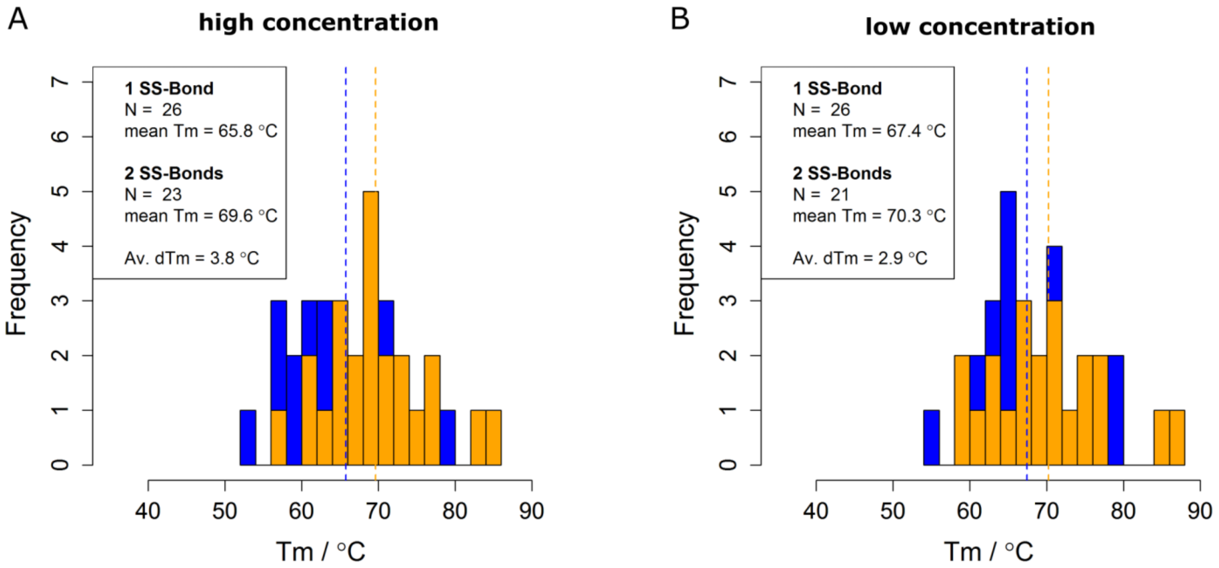

Supplementary Figure 8: The second disulfide bond in dromedary-derived binders and its effect on thermostability. Melting points were measured at (**A**) 32.7 µM and (**B**) 13.1 µM nanobody concentration in PBS, pH 7.4. Separate groups of dromedary-derived nanobodies with one (blue) and two (orange) disulfide bonds are shown. Dashed lines indicate the location of the mean T_m value of the respective group. “Av.dTm” in the legend gives the difference in mean T_m values of both groups.

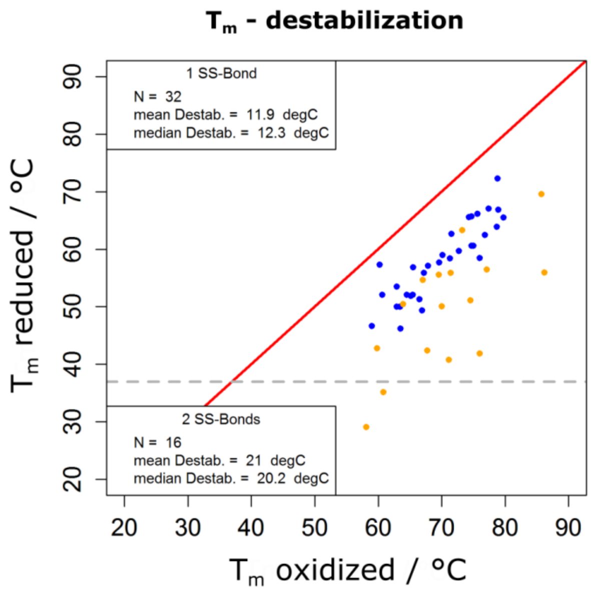

Supplementary Figure 9: Relation of T_m values of nanobodies with one (blue) and two (orange) disulfide bonds in presence and absence of 25 mM TCEP in PBS, pH 7.4. Gray dashed line indicates a temperature of 37 °C. T_m values of the large majority of nanobodies lie above 37°C.

Supplementary Tables

Supplementary Table 1: Aggregation rates and amplitudes at 32.7 µM nanobody concentration obtained from least-squares fitting and melting temperatures used for kinetic measurements in Figure 3A of the main text. Melting temperatures were obtained at a concentration of 13.1 µM in PBS, pH 7.4. Aggregation traces in Figure 3A were fitted where possible using the equation $\boldsymbol{F(t) = A * (1 - \exp(-k_{app*t}))}$, with the amplitude $\boldsymbol{A}$ and the apparent rate constant of aggregation $\boldsymbol{k_{app}}$. Values in brackets indicate 95% confidence intervals.

Nanobody	T_m (°C)	amplitude	rate constant k (s^{-1})	R-square
NbD3	67.2	93.0 (89.1; 96.8)	0.736 (0.564; 0.909)	0.99
NbSTK38L_1	74.3	89.6 (82.5; 96.7)	0.538 (0.334; 0.741)	0.99
NbSH2D2A_1	68.6	45.5 (37.9; 53.1)	1.292 (0.606; 1.978)	0.93
NbD1	65.4	75.1 (61.3; 88.9)	0.0345 (0.0185; 0.0536)	0.98
NbTDT_2	60.4	0.73 (-34.92; 36.38)	0.0027 (-0.1399; 0.1454)	0.52
NbTDT_4	76.7	9.37 (-1495; 1514)	0.0003 (-0.0498; 0.0505)	0.91
